# Dr. Frederick Erastus McClellan

**Published:** 1915-05

**Authors:** 


**Dr. Frederick Erastus McClellan,** Buffalo, 1890, died at his home in Canandaigua, from heart disease, Mch. 27, aged 46. He was a member of the staff of the Thompson Memorial Hospital.

